# Avoiding the Learning Curve for Transcatheter Aortic Valve Replacement

**DOI:** 10.1155/2017/7524925

**Published:** 2017-01-26

**Authors:** Sergey Gurevich, Ranjit John, Rosemary F. Kelly, Ganesh Raveendran, Gregory Helmer, Demetris Yannopoulos, Timinder Biring, Brett Oestreich, Santiago Garcia

**Affiliations:** ^1^University of Minnesota-Fairview Medical Center, Minneapolis, MN, USA; ^2^Minneapolis VA Healthcare System, Minneapolis, MN, USA

## Abstract

*Objectives.* To evaluate whether collaboration between existing and new transcatheter aortic valve replacement (TAVR) programs could help reduce the number of cases needed to achieve optimal efficiency.* Background.* There is a well-documented learning curve for achieving procedural efficiency and safety in TAVR procedures.* Methods. *A multidisciplinary collaboration was established between the Minneapolis VA Medical Center (new program) and the University of Minnesota (established program since 2012, *n* = 219) 1 year prior to launching the new program.* Results.* 269 patients treated with TAVR (50 treated in the first year at the new program). Mean age was 76 (±18) years and STS score was 6.8 (±6). Access included transfemoral (*n* = 35, 70%), transapical (*n* = 8, 16%), transaortic (*n* = 2, 4%), and subclavian (*n* = 5, 10%) types. Procedural efficiency (procedural time 158 ± 59 versus 148 ± 62, *p* = 0.27), device success (96% versus 87%, *p* = 0.08), length of stay (5 ± 3 versus 6 ± 7 days, *p* = 0.10), and safety (in hospital mortality 4% versus 6%, *p* = 0.75) were similar between programs. We found no difference in outcome measures between the first and last 25 patients treated during the first year of the new program.* Conclusions.* Establishing a partnership with an established program can help mitigate the learning curve associated with these complex procedures.

## 1. Introduction

The introduction of a novel technology is usually accompanied by a period of learning in which operators develop and refine new skills until they achieve a “steady state” characterized by high efficiency and procedural success with low complications [1, 2]. In the Placement of Aortic Transcatheter Valve (PARTNER) trial, 26 cases were required to achieve a sustained level of procedural performance and safety profile with transfemoral (TF) transcatheter aortic valve replacement (TAVR) [3].

The number of new TAVR procedures in the US has doubled in fiscal year (FY) 2013 from the previous year, with 10,599 total Medicare claims in FY 2013 as compared to 5,400 claims in FY 2012 [4]. The number of TAVR programs is also rapidly proliferating, from 228 in FY 2012 to 336 in FY 2013. The results of PARTNER 2A and SAPIEN 3 registry in intermediate-risk patients suggest that these trends will accelerate in the near future as TAVR indications expand to lower risk patients [5, 6].

In this context, it is important to develop strategies and partnerships that allow initiation of new TAVR programs that can produce efficacy and safety results that are comparable to existing national benchmarks in a reasonable period of time. In this manuscript, we describe our experience during the launch of a new TAVR program at the Minneapolis VA Medical Center. This program was launched after 1 year of close collaboration, proctorship, and hands-on experience with the University of Minnesota Medical Center.

## 2. Methods

The Minneapolis VA Healthcare System (MVAHCS) is a tertiary, 250-bed hospital within the VA Midwest Heath Care Network (Veterans Integrated Service Network VISN 23). The network serves more than 440,000 enrolled Veterans residing in the states of Iowa, Minnesota, Nebraska, North Dakota, and South Dakota and portions of Illinois, Kansas, Missouri, and Wyoming. The MVAHCS is the only approved TAVR program in an eight-state area and has an academic affiliation with the University of Minnesota. The University of Minnesota Medical Center (UMMC) has an existing TAVR program since 2012 and had performed 140 TAVR procedures prior to mentoring the MVAHCS program.

### 2.1. Mentorship Plan

Prior to launching the TAVR program, an institutional collaboration agreement was established between MVAHCS (new program) and UMMC (established program) with the goal of launching the new program with high success and low complications rates that were comparable to national benchmarks.

Specific interventions included the following: (1) common weekly TAVR video conference to discuss patients by a multidisciplinary team of interventional cardiologist, cardiac surgeons, anesthesiologists, and cardiac imaging, (2) privileging and hands-on training of lead interventional cardiologist and cardiac surgeon (total of 20 cases each, 7 as second operator and 13 as primary), (3) observation of UMMC cases by MVAHCS heart team members including anesthesiologists, perfusionists, and operating room and cardiac catheterization laboratory personnel, and (4) sharing of order sets and imaging protocols (i.e., CTA for annular sizing).

### 2.2. Patients and Outcomes

We included 219 patients treated with TAVR at UMMC since 2012 and 50 patients treated at MVAHCS during the first year of the program (April 2015–April 2016). We excluded patients that underwent transcatheter valve replacement in a nonaortic position (i.e., mitral valve in valve procedures or pulmonary). Patients that underwent TAVR procedures for off-label indications (bicuspid valve and aortic insufficiency) and/or valve in vale (VIV) procedures were included in the analysis.

Outcomes measures included procedural efficiency, as assessed by procedural time and contrast volume. Contrast volume included contrast used for peripheral angiography at the end of the procedure, which is routinely used in both programs. Procedural time was the time from arterial puncture or surgical incision until the end of the procedure. Measures of procedural success (device success) and safety (stroke, vascular complications, pacemaker requirement, and in-hospital mortality) were assessed in both groups using Valve Academic Research Consortium (VARC) definitions [7].

### 2.3. Statistical Methods

Comparisons between groups were made using Student's *t*-test (expressed as mean value standard deviation) for continuous variables. Indices were tested for normality of distribution, with nonnormally distributed data compared using 2-sample *t*-tests after initial logarithmic transformation. Categorical variables were compared using chi-square or, when there are fewer than 5 expected outcomes per cell, Fisher's exact test. Two-sided *p* < 0.05 was considered indicative of statistical significance. Statistical calculations were performed using STATA Statistics Version 12 (StataCorp LP, College Station, Texas).

## 3. Results

The study population consisted of a total of 269 patients undergoing transcatheter aortic valve replacement at UMMC and MVAHCS between 2012 and 2015. The mean age of the patients (*n* = 50) treated in the new program during the first year was 79 (±8) and the mean Society of Thoracic Surgeons (STS) risk score was 6.8. Transfemoral (TF) access was used in 70% and alternative access in the remaining 30%. Alternative access included transapical (*n* = 8), transaortic (*n* = 2), and subclavian (*n* = 5) types. Of the TF cases at the established program, 90 were percutaneous and 43 used a cut-down approach. Most cutdowns were used during 2012-2013, with first-generation valves. At the new program, all TF cases were percutaneous. At the established program, general anesthesia was used in all cases but 7 which were done with moderate sedation, and 1 was epidural. All of the cases at the new program were done with general anesthesia. A 12% increase, from 64% to 76%, in TF access was seen in the second half of the year (*p* = 0.36). A balloon-expandable valve (SAPIEN XT or SAPIEN 3, Edwards Life Sciences, Irvine, CA) was used in 80% of cases and a self-expandable valve (Corevalve or Evolut R, Medtronic, Minneapolis, MN) in the remaining 20%. The average valve size was 27.7 (±2) mm. The mean age of the patients (*n* = 219) treated in the established program was 81.5 (±9) and the mean STS score was 8. Patients in the established program had a higher prevalence of hypertension, heart failure, and previous myocardial infarction (Table 1). A total of 14 VIV procedures were performed in the established program and 8 in the new program. Other baseline characteristics were similar as outlined in Table 1. Individual operator experience is provided in the Supplementary Appendix in Supplementary Material available online at https://doi.org/10.1155/2017/7524925.

### 3.1. Procedural Efficiency

**Figure 1:**
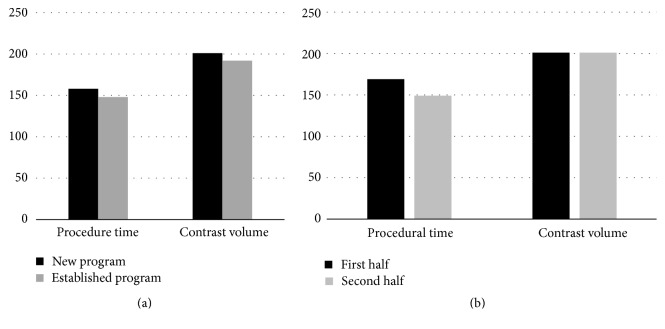
Procedural efficiency of new TAVR program relative to established program (a) and comparison of results of first versus second half of the first year of the new TAVR program.

Overall procedure time was 158 ± 59 minutes for the new program and 148 ± 62 minutes for the established program (*p* = 0.27) (Table 2). Average procedure time was 20 minutes lower for the last 25 patients treated in the new program relative to the first 25 cases, but these differences were not statistically significant (*p* = 0.26) (Figure 1). Contrast volume was 201 (±114) mL in the new program and 192 (±111) mL in the established program (*p* = 0.61) (Table 2). Contrast utilization remained unchanged during the first and second half of the first year of the new program (201 ± 136 mL versus 201 ± 88 mL, *p* = 0.5).

### 3.2. Procedural Outcomes

**Figure 2:**
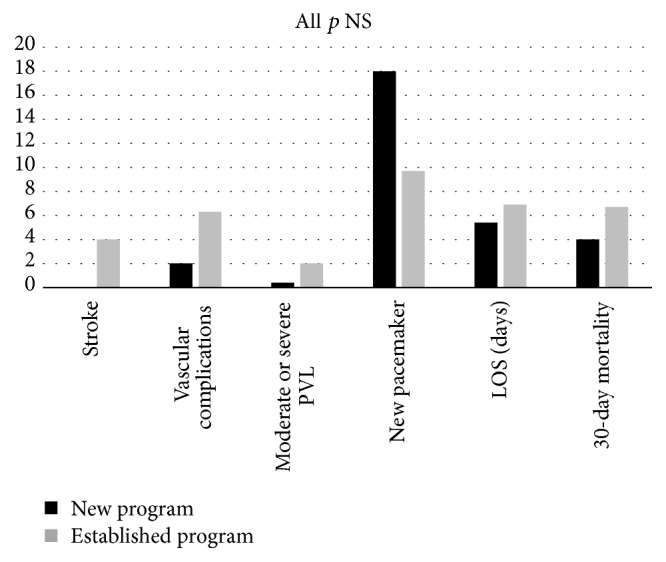
Procedural complications, length of stay, and in-hospital mortality.

Device success was high, 96% for the new program and 87% for the established program (*p* = 0.08) (Table 2). In the established program, 19 device failures occurred including 14 that required a second valve, 2 aborted due to access complication, 2 converted to valvuloplasty, and 1 valve embolization into the left ventricle. In the new program, there were 2 device failures, and both required a second valve (Table 2). Serious procedural complications were infrequent during the first year of the new program: vascular complications 2%, paravalvular leak moderate/severe 2%, and no strokes (Table 2). A permanent pacemaker was required in 18% of patients treated during the first year of the new TAVR program (Table 2). A trend toward reduction in pacemaker requirement was seen in the second half of the year (8%) relative to the first half (26%). In-hospital mortality was 4% and length of stay was 5 (±3 days) (Table 2). A comparison of procedural outcomes with the established program is presented in Figure 2 (all *p* = NS).

## 4. Discussion

There are several significant findings from this study. First, a new TAVR program launched in close collaboration with an existing TAVR program was associated with low complication rates and high device success from its inception. Second, procedural efficiency and length of stay were similar between the existing and the new TAVR programs. Third, mortality at 30 days was low at 4%, which is similar to the most recent STS/ACC TVT registry report [8]. Fourth, a learning curve previously described for TAVR procedures in the PARTNER trial was not observed in our series despite high-procedural complexity (nontransfemoral valve delivery in 30% of cases).

TAVR procedures are highly complex and require a multidisciplinary heart team approach between medical and surgical disciplines to achieve optimal outcomes [9]. A learning curve has been well-documented in the PARTNER trial as well as in single center experience [3, 10, 11]. Alli et al. reported their experience with the first 44 patients treated at the Mayo Clinic using a TF approach [10]. With increased experience, they showed significant reductions in contrast volume and time from valvuloplasty to valve deployment with evidence of plateau after 30 cases. Similarly, in the PARTNER I trial, Minha et al. showed that it took an average of 28 cases to achieve a consistently low risk of 30-day major adverse events for institutions entering the trial early. Interestingly, centers that came into PARTNER late into the trial were able to shorten the learning curve to 26 cases, which suggest they benefited from the experience gained and shared by existing programs [11]. It should be noted that both the Mayo Clinic and PARTNER I trial description of learning curve with TAVR procedures is restricted to TF access. Our series include 30% of patients treated with alternative access, mostly TA but also subclavian and transaortic. The learning curve for TA access is steeper with 30–45 cases required to achieve optimal procedural efficiency [12].

In the United States, the number of TAVR programs enrolled in TVT registry has more than doubled from 156 in 2012 to 348 in 2014 [8]. Given that the majority of surgical aortic valve replacement (SAVR) procedures are performed in lower risk patients [13], it is expected that this trend of growth in the number of TAVR programs will continue or even accelerate in the near future. Our experience of close collaboration with an existing TAVR program demonstrates that the learning curve associated with these procedures can be mitigated by training, proctorship, and sharing of best practices. This model of collaboration could be used as template for other TAVR programs.

## 5. Limitations

Our study has several limitations. First, we recognize that most VA Medical Centers have an academic affiliation with Universities and this may not be the case for other programs in the private sector. Competition for market share, limited time for physician training, and obtaining hospital privileges in multiple institutions could be important barriers to collaboration. Second, we are not able to identify which aspect of the training was more important. Third, reductions in the size of the delivery system and enhanced valve performance have significantly simplified the procedure as reflected by increased adoption of conscious sedation [14]. Future valve enhancements may reduce the need for intense training and proctorship. Finally, comparator cohort includes historical learning curve with utilization of first-generation valves and low device success rate.

## 6. Conclusions

We found that establishing a partnership with an existing TAVR program mitigated the learning curve associated with these complex procedures. The new program had similar outcomes to established programs in the US since its inception.

This collaborative model could be used by other institutions planning to become TAVR centers.

## Supplementary Material

Included in the Supplementary Appendix are the individual operator volumes of the established and new programs. Also included are operator volumes at the new program prior to starting the program.

## Figures and Tables

**Table 1:** Baseline characteristics.

Parameter	Established program overall (*n* = 219)	New program overall (*n* = 50)	*p*	New program		*p*
				First half (*n* = 25)	Second half (*n* = 25)	
*Clinical characteristics*						
Age (years)	81.4 ± 9.1	78.9 ± 8.7	0.08	79 (±8)	78 (±9)	0.47
Male gender	53% (115)	100% (50)	<0.01	100%	100%	NA
STS Score	8.0 ± 4.7	6.8 ± 6.0	0.15	6.2 ± 5	7.4 ± 7	0.25
Height (cm)	167 ± 11	172 ± 7.4	<0.01	171 ± 7.5	173 ± 7.4	0.57
Weight (kg)	81 ± 24	92 ± 25	<0.01	93 ± 31.5	91 ± 17	0.84
Ejection fraction	52.9 ± 12.1	50.3 ± 11.2	0.15	51.7 ± 11.2	48.8 ± 11.2	0.37
Heart failure	33% (73)	12% (6)	<0.01	0% (0)	24% (6)	0.02
Myocardial infarction	28% (61)	15% (7)	<0.01	8% (2)	21% (5)	0.42
PCI	36% (79)	35% (17)	0.86	20% (5)	50% (12)	0.04
Atrial fibrillation	44% (97)	42% (21)	0.77	40% (10)	44% (11)	0.78
Stroke	15% (32)	16% (8)	0.76	4% (1)	29% (7)	0.02
Porcelain aorta	6% (14)	4% (2)	0.74	8% (2)	0% (0)	0.49
Pacemaker	13% (29)	10% (5)	0.64	8% (2)	12% (3)	1.00
Smoker	7% (16)	6% (3)	1.00	4% (1)	8% (2)	1.00
Hypertension	89% (195)	52% (26)	<0.01	52% (13)	52% (13)	1.00
Dialysis	5% (10)	12% (6)	0.05	8% (2)	16% (4)	0.67
PAD	33% (72)	24% (12)	0.25	24% (6)	24% (6)	0.94
Diabetes mellitus	33% (73)	32% (16)	0.31	36% (9)	28% (7)	0.83
Home oxygen	13% (28)	22% (11)	0.10	24% (6)	20% (5)	1.00
Anticoagulant	19% (33)	10% (5)	0.15	8% (2)	12% (3)	1.00
*Aortic valve measurements*						
Area	0.82 ± 0.52	0.76 ± 0.23	0.43	0.72 ± 0.22	0.80 ± 0.23	0.27
Peak velocity	4.2 ± 0.69	3.9 ± 0.68	0.01	4.1 ± 0.7	3.7 ± 0.6	0.03
Mean gradient	43.9 ± 14	38.2 ± 14.2	0.01	42.2 ± 16.5	34.4 ± 10.6	0.05
*Procedural characteristics*						
Transfemoral access	61% (138)	70% (35)	0.25	64%	76%	0.36
Alternative access	39% (87)	30% (15)	0.25	36%	24%	0.36
Balloon-expandable	79% (171)	80% (40)	0.61	84%	76%	0.73
Self-expandable	21% (52)	20% (10)	0.61	16%	24%	0.73
Valve size (mm)	26 ± 2.5	27.7 ± 2.2	<0.01	27.5 (±2)	27.9 (±2)	0.40

**Table 2:** Procedural efficiency, device success, and safety.

Parameter	Established program overall (*n* = 219)	New program overall (*n* = 50)	*p*	First half (*n* = 25)	Second half (*n* = 25)	*p*
*Procedural efficiency*						
Contrast (cc)	192.4 ± 111.2	201.3 ± 114.7	0.61	201 (±136)	201 (±88)	0.5
Procedure time (min)	148 ± 62.7	158.9 ± 59.1	0.27	169 ± 62.7	149.6 ± 55.2	0.26
*Procedural success and safety*						
Device success	87.2%	96%	0.08	96%	96%	0.5
Stroke	4.0% (9)	0% (0)	0.37	0%	0%	—
Vascular complications	6.3% (14)	2% (1)	0.32	0%	1 (4%)	0.29
Paravalvular leak(>mild)	0.4% (1)	2% (1)	0.34	4%	0%	1.00
Pacemaker placement	9.7% (21)	18% (9)	0.10	7 (26%)	2 (8%)	0.08
Length of stay (days)	6.9 ± 7.5	5.42 ± 3.5	0.17	6 (3)	5.3 (3)	0.7
In-hospital mortality	6.7% (15)	4% (2)	0.75	1 (4%)	1 (4%)	0.50
