# Supplementary material for: Avoiding the Learning Curve for Transcatheter Aortic Valve Replacement
Source: Cardiol Res Pract. 2017 Jan 26;2017:7524925. doi: 10.1155/2017/7524925 (PMC5299191; doi:10.1155/2017/7524925)
Supplement: Supplementary file 1 — Included in the Supplementary Appendix are the individual operator volumes of the established and new programs. Also included are operator volumes at the new program prior to starting the program. [file 7524925.f1.docx]

***Supplementary Appendix: Individual operator experience***

***Established Program****:*

Operator A: 110 (interventional cardiologist)

Operator B: 211 (interventional cardiologist)

Operator C 53 (cardiac surgeon)

Operator D 38 (cardiac surgeon)

***New Program:***

*Prior to launching:* total of 20 cases each, 7 as second operator and 13 as primary

*During first year of the program:*

Operator A: 50 (interventional cardiologist)

Operator B: 15 (cardiac surgeon)
